# Use of selected ambulatory dental services in Taiwan before and after global budgeting: a longitudinal study to identify trends in hospital and clinic-based services

**DOI:** 10.1186/1472-6963-12-339

**Published:** 2012-09-25

**Authors:** Chienhung Lin, Hailun Chao

**Affiliations:** 1 Department of Health Care Administration, Chung-Hwa University of Medical Technology, Tainan, Taiwan

**Keywords:** Global budgeting, National health insurance, Taiwan, Dental services

## Abstract

**Background:**

The Taiwan government adopted National Health Insurance (NHI) in 1995, providing universal health care to all citizens. It was financed by mandatory premium contributions made by employers, employees, and the government. Since then, the government has faced increasing challenges to control NHI expenditures. The aim of this study was to determine trends in the provision of dental services in Taiwan after the implementation of global budgeting in 1998 and to identify areas of possible concern.

**Methods:**

This longitudinal before/after study was based on data from the National Health Insurance Research Database from 1996 to 2001. These data were subjected to logistic regression analysis. Linear regression analysis was used to examine changes in delivery of specific services after global budgeting implementation. Utilization of hospital and clinic services was compared.

**Results:**

Reimbursement for dental services increased significantly while the number of visits per patient remained steady in both hospitals and clinics. In hospitals, visits for root canal procedures, ionomer restoration, tooth extraction and tooth scaling increased significantly. In dental clinics, visits for amalgam restoration decreased significantly while those for ionomer restoration, tooth extraction, and tooth scaling increased significantly. After the adoption of global budgeting, expenditures for dental services increased dramatically while the number of visits per patient did not, indicating a possible shift in patients to hospital facilities that received additional National Health Insurance funding.

**Conclusions:**

The identified trends indicate increased utilization of dental services and uneven distribution of care and dentists. These trends may be compromising the quality of dental care delivered in Taiwan.

## Background

The government of Taiwan adopted National Health Insurance (NHI) on March 1, 1995 in order to provide universal health care to all its citizens. This comprehensive single-payer, fee-for-service health benefit coverage is financed by mandatory premium contributions made by employers and employees, and NHI-related tax revenues from the government. The system is run by an agency of the central government’s Department of Health, the Bureau of National Health Insurance (BNHI) [1]. Within a year of establishing the program, healthcare utilization rates of Taiwan’s uninsured population was at the level of the previously insured population and by 2003, coverage was more than 96% [1].

Since the introduction of NHI, BNHI has faced increasing challenges to control its expenditures. The increase in the general population, disproportionate increase in the percentage of elderly in the population, advances in medical technology, rising incomes associated with increased healthcare expectancy by the population, and the increase in utilization engendered by universal, comprehensive benefit coverage have all contributed to increasing healthcare expenditures [1,2]. As a result, NHI expenditure has progressively outstripped its income from premiums and co-payments. According to the Directorate General of Budget Accounting and Statistics, 2004, nominal per capita health expenditures increased seven-fold between 1980 and 2000, from NT$ 2,805 (New Taiwanese dollars) to NT$ 23,419, which, adjusted for general inflation amounted to a 69% increase.

To limit the escalating medical expenditures induced by this fee-for-service system of reimbursement, Taiwan implemented a global budgeting system on July 1, 1998. This system, modeled in some respects after a German system that stabilized the national insurance contribution rate [3], included dental care in its scope of services and dentists were the first providers to be subject to global budgeting policies. A “global budget” is based on the annual growth rate of the insured populaton and is influenced by effects of changing population on dental care (or other healthcare services) expenditures and changes in the dental care cost index, with growth indicators such as weighted values of salary and/or price index [1,4,5]. Other factors that influence the global budget are effects of changing insurance coverage on dental care expenditures, growth rates of insurance premiums, growth in the number of dentists and variables such as quality of healthcare services and economic growth reflecting ability to pay [1,5]. Most national health insurance systems that employ global budgeting do not typically include dental services [4]. Global budgeting is considered a radical cost control measure; it establishes a budget for providers and requires them to deliver healthcare to their entire patient population without exceeding that budget [5]. The German system [3], the UK system [5] and the Taiwan system [1] have implemented global budgeting to control cost inflation associated with fee-for-service systems. However, the differences in Taiwan’s global budgeting system include that it is a single-payer system with an expenditure cap and a regional budgeting system [1,6]. Six regional budgets are allocated based on their proportion of the insured population, adjusted progressively year to year. Healthcare providers in each region are reimbursed by fee-for-service point values that are calculated retrospectively every three months. Therefore, quarterly payments for the regions are based on floating point values. Taiwan’s single-payer NHI system has been credited with having kept health spending inflation under control and the related savings are reported to have offset the added cost of covering previously uninsured citizens [6]. A recognized problem with global budgeting, however, is that it tends to encourage rationing of care at the system or policy level, which could manifest as limiting technology coverage or delaying new technology introductions as it has in the UK, or delaying access to the latest drugs by 2 to 5 years as it has in Taiwan; however, Taiwan has built cost containment into its system [4] and both Taiwan and the UK have experienced significantly less healthcare inflation than the United States under its Medicare system [5]. While global budgeting systems control inflation, it remains to be seen *if* and *how* quality of care may be affected. The results of Chang and Hung [7] suggest that there may be a trade-off effect by which cost containment comes at the expense of healthcare quality.

Successful implementation and management of global budgeting requires that the BNHI and healthcare providers negotiate the volume of total expenditure and its fair distribution. The BNHI delegated decision-making to the dental associations for financial allocations within regions, subject to the limit set by the global budget.

The trends in dental care are relatively easy to monitor since dentists only treat outpatients, perform a limited number of procedures, and do so repeatedly. In Taiwan, dental services are provided in two major venues: hospitals (6%) and clinics (94%). The healthcare facilities in Taiwan are primarily privately owned, including 86% of hospitals and 65% of hospital beds [1]. Doctors and dentists are either salaried hospital staff (63%) or they are self-employed, fee-for-service practitioners in their own clinics (37%) [6]; 97% of clinics are privately owned [1]. Taiwan’s healthcare system is market driven owing to the free-enterprise system and the number of private facilities [6], and competition for patients is extensive, especially in a high capacity healthcare system like Taiwan’s where capacity may exceed volume of utilization [1,2]. Hospitals do not fill all their beds, of which 35% are public and 65% private, and under NHI they have begun to develop large outpatient services and clinics to help maintain their flow of inpatients [6]. As a result of the imbalance in private and public healthcare services and the ensuing competition for patients in the global budgeting system, the healthcare delivery system is generally fragmented and lacks continuity of care [6]. These reports suggest that we must ask how the NHI and global budgeting may have affected dental care services—and the costs and quality of dental care.

The aims of this study were to determine changes in the types and locations of services provided after the implementation of global budgeting and to identify areas of potential concern about the trends in dental services in Taiwan.

## Methods

### Study design and data collection

This longitudinal before/after study analyzed data from the National Health Insurance Research Database (NHIRD; http://www.nhi.gov.tw.) and monthly bulletins from the Department of Statistics, Ministry of the Interior concerning Bureau of National Health Insurance (BNHI) outpatient reimbursement for dental care provided during 1996 to 2001. The NHIRD maintains anonymity of all NHI enrollees whose data are included in the database, precluding the need for signed informed consent from individual subjects. The study protocol was reviewed and approved by the Institutional Review Board of Chung-Hwa University of Medical Technology, Tainan, Taiwan.

### The national health insurance research database

The automated NHIRD includes data of 23 million Taiwanese citizens, covering 99% of Taiwan’s population, including that of all geographic regions and off-shore islands [8]. Enrollees are predominantly employer-based but also include low income and disabled individuals. The BNHI cooperates with the National Health Research Institute to establish and administrate the NHI research databases, making it available to scholars through contractual agreements with hospitals, clinics, academic medical centers and health-related institutions and organizations. Abundant information on demographics, medical services utilization and related costs is made available through inpatient and outpatient medical records, including subgroup populations such as children, pregnant women, and older adults (>age 65 years) [8]. Twelve databases are available, addressing diabetes, cancer, psychology, case payment and others. The NHIRD included three databases relative to dental care: a registry for contracted dental facilities, monthly claim summaries for ambulatory care claims, and details of ambulatory care orders. The registry for contracted dental facilities provides data on the following items: contracted beds, contracted specialty services, board-certified specialists, and dental personnel. It also provides data on ownership of the dental institutions (public or private), accreditation level (medical center, regional hospital, community hospital, and clinic), and type of institution (hospital or clinic). Monthly summaries for ambulatory care claims contain outpatient reimbursement fees, number of reported cases, and treatment duration for each claim. Ambulatory care order information includes case-wise ambulatory care expenditures by visit and procedure.

### Statistical analysis

Independent two sample t tests were used to compare the indices of dental care practices within institutional types before and after global budgeting. The following dental care variables were analyzed: average reimbursement fee, average reimbursement fee per visit, average reimbursement fee per patient, and average reimbursement fee per dentist. Linear regression analysis was used to examine the changes in likelihood of specific treatments after global budgeting implementation. Residual analysis was used to check whether the distribution of residues abided by normality, consistency, and independence. The statistical analyses were performed using SAS 9.0 (SAS Institute Inc., Cary, NC, USA), and the significance level was set at 0.05.

## Results

The annual changes in dental care statistics between 1996 and 2001 are presented in Table 1. Access to dental care, indicated by the total number of patients treated, improved significantly over the period. Increased financial accessibility through insurance coverage also affected the total number of visits and the dental reimbursements, both of which rose annually throughout the study period. Despite these increases, the number of visits per patient, the reimbursements per dental visit, and the reimbursements per patient remained stable over the same period. In fact, the number of dental visits per patient declined after 1998. The number of dentists increased from 7,254 in 1996 to 8,944 in 2001, for an overall increase of 23.3%. In 2001, there were 3.99 dentists per 10,000 people.

**Table 1:** Number and percent of total reimbursements, visits, patients, dentists and their interactions, 1996-2001

	**Before global budgeting**			**After global budgeting**		
	**1996**	**1997**	**1998**	**1999**	**2000**	**2001**
Total patients	11,607,374	12,909,396	13,715,022	14,309,879	14,685,823	15,520,014
Total dentists	7254	7573	7900	8240	8597	8944
Total visits	19,067,638	21,499,247	22,912,081	23,482,032	23,784,239	24,802,145
Reimbursements^*^	18,080,222,820	20,674,781,728	22,890,141,602	24,399,749,172	26,310,857,132	27,954,926,509
Visits/patient	1.64	1.67	1.67	1.64	1.62	1.60
Reimbursements/visit^*^	948.22	961.65	999.04	1,039.08	1,106.23	1,127.12
Reimbursements/patient^*^	1,557.65	1,601.53	1,668.98	1,705.10	1,791.58	1,801.22
Reimbursements/dentist^*^	2,912,289.75	3,143,337.99	3,387,660.78	3,431,549.84	3,483,958.84	3,533,343.00
Dentists/10,000 people	3.37	3.48	3.60	3.73	3.86	3.99

* in NT$.

Global budgeting had a statistically significant impact on increasing reimbursement. Table 2 shows reimbursements and their relationship to visits, patients, and dentists in different types of dental institutions. With regard to total reimbursement, the mean was NT$ 1,650,918,398 per month in the pre-global budget period and NT$2,161,503,024 per month in the post-global budget period (P < 0.001). There were significant differences in reimbursement per visit, reimbursement per patient and reimbursement per dentist before and after global budgeting (all P < 0.001). In terms of total visits, 7.76% were in hospitals and 92.24% in clinics from 1996 to 2001. Reimbursement to hospitals was NT$129,955,704 per month in the pre-global budget period and NT$ 170,655,657 in the post-global budget period (p=1.00). The reimbursement per visit was NT$ 968.29 in the pre-global budget period and NT$ 1,101.35 in the post-global budget period (P < 0.001). In summary, hospital visits per patient decreased significantly, but reimbursement per patient and reimbursement per visit increased significantly after global budgeting. In clinics, the amount of reimbursement was NT$1,520,962,694 in the pre-global budget period and NT$ 1,990,847,336 in the post-global budget period (P < 0.001). For clinics, the total reimbursement, reimbursement per visit, reimbursement per patient, and reimbursement per dentist increased significantly after global budgeting.

**Table 2:** Reimbursements and their relationship to visits, patients, and dentists in different types of dental institutions (in NT$)

	**Before global budgeting**		**After global budgeting**		**P value**
	**Mean**	**SE**	**Mean**	**SE**	
**Total**					
Reimbursements	1,650,918,398	35,919,039	2,161,503,024	30,357,128	<0.001*
Visits/patient	1.65	.0069	1.63	.0059	.0027
Reimbursements/visit	959.30	6.85	1,080.51	5.79	<0.001*
Reimbursements/patient	1,587.50	10.86	1,756.54	9.18	<0.001*
Reimbursements/dentist	238,025.51	3,589.00	277,947.95	3,033.26	<0.001*
**Hospitals**					
Reimbursements	129,955,704	23,245,238	170,655,657	19,645,812	1.000
Visits/patient	1.58	.0074	1.54	.0063	.0004
Reimbursements/visit	968.29	8.25	1,101.35	6.97	<0.001*
Reimbursements/patient	1,530.91	12.40	1,695.22	10.48	<0.001*
Reimbursements/dentist	192,848.26	3,558.90	210,170.06	3,007.82	0.002*
**Clinics**					
Reimbursements	1,520,962,694	23,245,238	1,990,847,336	19,645,812	<0.001*
Visits/patient	1.65	.0074	1.62	.0063	.0227
Reimbursements/visit	958.54	8.25	1,078.74	6.97	<0.001*
Reimbursements/patient	1,577.53	12.40	1,743.50	10.48	<0.001*
Reimbursements/dentist	257,109.09	3,558.90	293,156.65	3,007.82	<0.001*

* Significant differences before and after global budgeting, P < 0.05.

**Figure 1:**
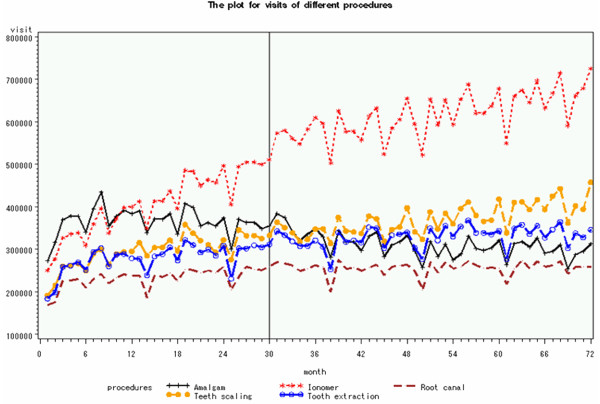
Visits for different dental procedures before and after global budgeting.

**Figure 2:**
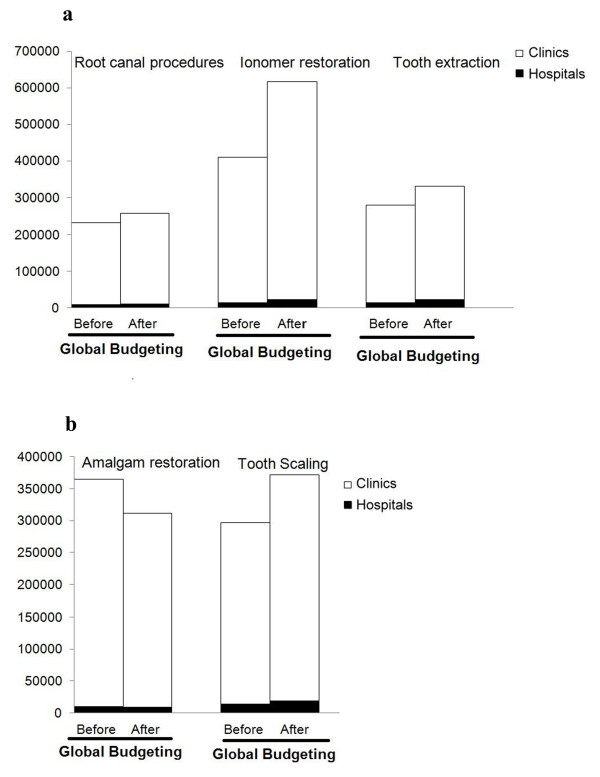
2a: High reimbursement; 2b: Low reimbursement.

Table 3 and Figure 1 show that total visits for ionomer restoration and tooth extraction achieved a significant increase after global budgeting. The visits for root canal procedures increased after global budgeting but did not reach significance. In the hospital subgroup, visits for root canal procedures, ionomer restoration, tooth extraction, and tooth scaling increased significantly after implementation of global budgeting. In dental clinics, the visits for amalgam restoration decreased significantly, but those for ionomer restoration, tooth extraction, and tooth scaling increased significantly after implementation of global budgeting (all P < 0.001) (Figure 2).

**Table 3:** Visits for different procedures in hospitals and clinics

**Procedures**	**Before global budgeting**		**After global budgeting**		**P value**
	**Mean**	**SE**	**Mean**	**SE**	
***High reimbursement***					
Root canal procedures					
Hospitals	7,678.53	453.21	10,399.05	383.04	<0.001*
Clinics	225,079.57	6,579.79	245,718.61	5,560.93	.77
Ionomer restoration					
Hospitals	13,906.67	453.21	22,809.48	383.04	<0.001*
Clinics	395,886.10	6,579.79	594,363.14	5,560.93	<0.001*
Tooth extraction					
Hospitals	14,059.87	453.21	22,664.88	383.04	<0.001*
Clinics	265,825.60	6,579.79	307,267.05	5,560.93	<0.001*
***Low reimbursement***					
Amalgam restoration					
Hospitals	9,853.47	453.21	8,646.88	383.04	1.00
Clinics	354,845.57	6,579.79	302,524.95	5,560.93	<0.001*
Tooth Scaling					
Hospital	282,849.10	6,579.79	352,667.83	5,560.93	<0.001*
Clinical	13,943.33	453.21	19,198.55	383.04	<0.001*

* Significant differences before and after global budgeting, P < 0.05.

Table 4 shows that the beta coefficients of the variable Time were positive and statistically significant across the models, indicating a general trend of increasing reimbursement for dental care in Taiwan. The beta coefficients for global budgeting implementation were also positive and statistically significant across the models as a result of improvement in reimbursement after the introduction of dental global budgeting. The interaction term was significant and positive in the model for reimbursement per visit and reimbursement per patient, as the reimbursement per visit and reimbursement per patient became more even over time in the post global budget period, despite a general time trend of increased reimbursement. The only significant and negative interaction term between Time and global budget implementation was associated with reimbursement per dentist.

**Table 4:** Parameter estimates of regression lines for reimbursement

**Variable**	**Total reimbursement**		**Reimbursement/visit**		**Reimbursement/patient**		**Reimbursement/dentist**	
	**β**	**SE**	**β**	**SE**	**β**	**SE**	**β**	**SE**
Intercept	1,388,940,295*	50,895,691.00	943*	5.56	1534*	16.98	246*	6.47
Time	16,886,632*	2,866,889.00	1*	0.31	3*	0.96	1*	0.36
Global budgeting implementation	524,929,280*	68,780,919.00	53*	7.51	134*	22.95	40*	8.75
Time x global budgeting implementation	−5,377,453	3,601,921.00	3*	0.39	1*	1.20	−2*	0.46
R^2^	0.8094		0.9509		0.7944		0.5222	

β: Parameter Estimate, * P < 0.05.

## Discussion

After the introduction of global budgeting in Taiwan, the total reimbursement for dental services, reimbursement per visit, reimbursement per patient, and reimbursement per dentist all increased significantly while the number of visits per patient decreased slightly. The number of high reimbursement procedures such as root canals, ionomer restoration, and tooth extractions increased while the number of low reimbursement amalgam restorations decreased. Nevertheless, although tooth scaling is a low reimbursement procedure, the number of tooth scalings increased.

Increases in utilization of certain services after universal NHI coverage and global budgeting can be explained in part by the overall increase in patient dental visits. Other changes may be based on dentists’ attitudes regarding services and financial parameters. Increases in root canal procedures, which were less than for other procedures, may be the result of dentists avoiding performing this complicated, arduous procedure in favor of alternative measures such as extraction [9]. A survey found that 80% of dentists thought root canal was underpaid and not cost-effective [10,11]. In Taiwan, root canal procedures also have a more compex reimbursement process through BNHI. Tooth extraction, besides increases due to the overall increase in patient visits, has great variations in reimbursement based on number of teeth and their location, which may increase procedure complexity, thereby reducing dentists’ willingness to perform extractions in some cases. Dentists may also avoid extractions for children, uncooperative patients or those wih seizure, heart disease and poor health status. However, failure of root canal procedures may contribute to increased extractions. Ionomer restoration is preferred by dentists over amalgam restoration since the latter alters the appearance of teeth and harms the environment [12]. Well informed dentists and dental patients tend to choose ionomer restoration over amalgam restoration [13], which may help to explain the decrease of amalgam restoration in the present study. Tooth scaling is an essential preventive procedure and relatively easy to perform [14]. Dentists are willing to provide this service given the low relative risk, preventive potential and costs [13,15]. With regularly scheduled oral examination and teeth scaling, which increased during our study period, other procedures such as root canal and extraction generally decrease.

Results of the present study show that the number of dentists increased by 23.3% between 1996 and 2001 post-NHI and that more dentists did provide more services to more patients. However, a study of the effects of global budgeting on the distribution of dentists and the use of dental care indicates that global budgeting did not effectively improve the distribution and supply of dentists in Taiwan even though the NHI system with global budgeting is a redistributive system that sets budget caps for each healthcare region according to its population (per capita) [4]. Equity in dental care resources was an explicit goal of global budgeting in Taiwan and equitable financial access to all citizens was essentially achieved [4]. The present study showed that, although there were more dentists, per-patient utilization of their services decreased, but not significantly; changes in the number of dental visits and reimbursement amounts per patient were relatively small, which suggests that utilization control is indeed a factor of dental global budgeting. However, in the competitive market within the private sector where 90% of dentists are independent, *overall* utilization of dental services was reported to increase [16]. Similarly, a study of Blue Cross dental insurance claims in Ontario, Canada, reported an increase in the volume and intensity of services received by adult patients when fee constraints were imposed on the dentists, stressing the importance of fee schedule design and the billing practices of dental service providers [17].

The dental global budgeting process itself may have influenced the behavior of dentists. In a diffused market like Taiwan’s, the delivery of dental services may have a “trivial” impact on the payment rates of global budgets as previously described [4]. For example, dentists might not have performed procedures that did not yield proportionate levels of income in order to keep their service level in line with its income potential. There may also have been a tendency to shift procedures to public facilities that received additional funding from NHI. Bundling of services for preventive maintenance or recall services, which general dental practices stress as standard good care and which provide a base income, can vary from dentist to dentist; a study that examined the mix of bundled services concluded that this response of dentists to fee controls matched that of other medical markets and that fee schedule adjustments without accompanying regulations often leads to alterations in services offered to patients [17]. While dentists may bundle services to increase revenues within global budgeting, hospitals may admit more patients with discretionary conditions in order to increase revenue [18]. Although these trends may boost revenues of the individual practice or hospital, they may not effectively control expenditures of the system [17]. In this study, data suggest that some healthcare providers may have offered unnecessary services to a large number of patients in the original fee-for-service program, for which providers submitted related bills and received payment from the BNHI.

On the other hand, the BNHI has gradually decreased its subsidy to public hospitals and these facilities may have begun to outsource their dental services. The global budget system was implemented in Taiwan to help compensate for increasing losses and achieve cost containment for the NHI program and data from the present study and others indicate that it has done this to some extent. What still may be needed is additional focus on the per-capita allocation of budgets and an examination of system-wide outcomes and patients’ expenditures to determine if patients’ actual costs are associated with equitable quality care. Socio-economic and geographic factors need to be addressed in any evaluation and/or revision of the NHI, and insurance and visit types have the potential to be altered at the policy level to achieve better overall service outcomes, as suggested by other investigators [19].

Demand for services differs considerably in countries without a national insurance program. In Mexico, for example, where there is little public financing of dental care, an evaluation of dental expenditures and dental care decision making revealed that socio-economic factors, primarily household capacity to pay, governed whether or not dental care was sought [20]. Mexican citizens must pay out-of-pocket costs themselves and those who seek care do so in the private sector, pointing to inequities in Mexico’s healthcare financing. In Taiwan, where the NHI provides equal coverage to 99% of the population, we would expect uniform utilization of services and demand for care. However, because NHI reimbursement is calculated by health insurance regions with different allocated budgets based on demand and the practice patterns of dentists in the region, the improved access to dental care after NHI does not necessarily reduce the unequal distribution of dental care and dentist supply across the designated geographic regions [4]. Our data agree with this finding. We must consider that distribution of care and of dentists will improve as the system evolves but it will require staying within the mandated coverage guidelines and provision of incentives for dentists to relocate their practices and still maintain their intended income.

This study has several limitations, some due to trends that could not be evaluated using BNHI data. For example, this study lacks basic demographic information about patients and dentists and our data source did not distinguish certain differences such as rural vs. urban practices, male vs. female dentists, and younger vs. older dentists. It has been shown that relatively fewer dentists practice in rural areas [21]. A study by Lee and Jones [22] indicated that male and younger dentists showed greater policy effects than did female and older dentists. In the present study, the BNHI database from which all data were obtained does not differentiate between resident and attending dentists in hospitals or indicate if dentists practice in more than one location, which raises the possibility that related reimbursement figures may be inaccurate. Also, data about dentist reimbursement may be underestimated because out-of-pocket procedures such as dental crowns and implants are not covered by NHI and some dentists may have spent more of their time doing such procedures. Dishonest claims may also have produced false results. Other limitations included variations in the NHI payment for dental procedures that may differ from patient to patient. Payments for extraction, for example, depend on the number and location of the teeth, amalgam restoration on the number of faces, and ionomer restoration on the materials in the ionomer. There was no measure of quality of service in this study and a study by Chang and Hung [7] indicated that cost containment comes at the expense of health care quality. Another unknown factor that might influence the number of visits would be the recall policy to encourage follow-up, especially if it differed in hospitals and clinics [23]. Future studies should address these limitations as well as carry the study forward from 2004 to take into account the shift toward population-based funding and to determine if the observed trends continue or if initial unmet needs created only a temporary increase in the number and kind of services provided. The current system provides care to more patients but the question as to whether it is most efficient for those patients and most cost effective for the government remains unanswered.

## Conclusions

In summary, although our data show that access to dental care, financial accessibility through insurance coverage, and the number and amount of reimbursements increased after Taiwan adopted a national healthcare program and a global budgeting scheme, implementation of the new system has altered the way dental care is being delivered. The altered aspects include increases in the number of dentists and changes in their distribution; decreases in the number of dental visits per patients; a shift from hospital visits (decreased) to clinic visits (increased) as hospitals outsource certain dental services; and a decrease in performance of low-reimbursement procedures as dentists choose to perform more high-reimbursement procedures. These trends may suggest that some services and some patients fall into cracks in the system and that the quality of dental care delivered in Taiwan is being compromised. Additional research is needed to confirm the emergence of trends and related consequences.

## Competing interests

The author has no conflicts of interest to declare.

## Authors’ contributions

The author (HC) conceived of and conducted the study, analyzed all data and wrote the report. Statistical analyses were performed by a statistician.

## Pre-publication history

The pre-publication history for this paper can be accessed here:

http://www.biomedcentral.com/1472-6963/12/339/prepub

## References

[B1] Cheng TM Taiwan’s national health insurance Republic of China 2000 Health Statistical Trends 2002 Department of Health, Taiwan

[B2] Chang HJ Taiwan’s national health insurance: overview and perspectives Republic of China Health 2000 Health Statistical Trends 2002 Department of Health, Taiwan

[B3] Kamke K The German health care system and health care reform Health Policy 1998 43 171 194 10.1016/S0168-8510(97)00096-1 10177617

[B4] Hsueh YA Lee SD Huang YA Effects of global budgeting on the distribution of dentists and use of dental care in Taiwan Health Serv Res 2004 39 2135 2153 10.1111/j.1475-6773.2004.00336.x 15544648 PMC1361116

[B5] Goozner M Global budgeting: a viable alternative to fee-for-service health care? The Fiscal Times 2010 http://www.thefiscaltimes.com/Columns/2010/02/18/Global-Budgeting-A-Viable-Alternative-to Fee-for-Service-Health-Care.aspz. Accessed 15 Jul 2011

[B6] Lu JFR Hsaio WC Does universal health insurance make health care unaffordable? Lessons from Taiwan Health Aff 2003 22 77 88 10.1377/hlthaff.22.3.77 12757274

[B7] Chang L Hung JH The effects of the global budget system on cost containment and the quality of care: experience in Taiwan Health Serv Manage Res 2008 21 106 116 18482934 10.1258/hsmr.2008.007026

[B8] Hsiao FY Yang CL Huang YT Huang WF Using Taiwan’s national health insurance research databases for pharmacoepidemiology research J Food Drug Anal 2007 15 99 108

[B9] Brown AD Mellor AC Main JR A survey of patient perceptions of dental charge Prim Dent Care 1999 6 151 155 11819892

[B10] Oscarson N Källestal C Karlsson G Methods of evaluating dental care costs in the Swedish public dental health care sector Comm Dent Oral Epidemiol 1998 26 160 165 10.1111/j.1600-0528.1998.tb01944.x 9669593

[B11] Brown LJ Lazar V Trends analysis of dental expenditures by selected dentist and practice characteristics, 1985–1995 J Am Dent Assoc 1998 129 1615 1621 9818583 10.14219/jada.archive.1998.0112

[B12] Harris NO Garcia-Godoy F Primary Preventive Dentistry 2004 6 Pearson Prentice Hall, Upper Saddle River, NJ

[B13] Atchison KA Mayer-Oakes SA Schweitzer SO Lubben JE DeJong FJ Matthias RE The relationship between dental utilization and preventive participation amon a well-elderly sample J Pub Health Dent 1993 53 88 95 10.1111/j.1752-7325.1993.tb02681.x 8515416

[B14] Burt BA Eklund SA Dentistry, Dental Practice, and the Community 1999 5 WB Saunders, Philadelphia

[B15] Friedman JW Atchison KA The standard of care: an ethical responsibiity of public health dentistry J Pub Health Dent 1993 53 165 169 10.1111/j.1752-7325.1993.tb02696.x 8371195

[B16] Tsai WC Kung PT Chang WC Influences of market competition on dental care utilization under the global budget payment system Community Dent Oral Epidemiol 2007 35 459 464 10.1111/j.1600-0528.2006.00355.x 18039287

[B17] Porter J Coyte PC Barnsely Crawford R The effects of fee bundling on dental utilization Health Serv Res 1999 34 901 921 10536976 PMC1089048

[B18] Chen FJ Laditka JN Laditka SB Xirasanar Providers’ responses to global budgeting in Taiwan: what were the initial effects? Health Serv Manage Res 2007 20 113 120 10.1258/095148407780744624 17524223

[B19] Brennan DS Spencer AJ The role of dentist, practice and patient factors in the provision of dental services Community Dent Oral Epidemiol 2005 33 181 195 10.1111/j.1600-0528.2005.00207.x 15853841

[B20] Pérez-Núñez R Vargas-Palacios A Ochoa-Moreno I Medina-Solis CE Household expenditure in dental health care: national estimations in Mexico for 2000, 2002, and 2004 J Pub Health Dent 2007 67 234 242 10.1111/j.1752-7325.2007.00035.x 18087994

[B21] Allison RA Manski RJ The supply of dentists and access to care in rural Kansas J Rural Health 2004 23 198 206 17565519 10.1111/j.1748-0361.2007.00091.x

[B22] Lee MC Jones AM How did dentists respond to the introduction of global budgets in Taiwan? An evaluation using individual panel data Int J Health Care 2004 4 307 326 10.1023/B:IHFE.0000043760.57999.36 15467407

[B23] Nguyen L Hakkinen U Choices and utilization in dental care: public vs. private dental sectors, and the impact of a two-channel financed health care system Eur J Health Econ 2006 7 99 106 10.1007/s10198-006-0344-3 16489469

